# Determination and comparison of the smallest detectable change (SDC) and the minimal important change (MIC) of four-shoulder patient-reported outcome measures (PROMs)

**DOI:** 10.1186/1749-799X-8-40

**Published:** 2013-11-14

**Authors:** Derk A van Kampen, W Jaap Willems, Loes W A H van Beers, Rene M Castelein, Vanessa A B Scholtes, Caroline B Terwee

**Affiliations:** 1 Department of Orthopaedic Surgery and Traumatology, Waterland Hospital, Waterlandlaan 250, Purmerend 1441 RN, The Netherlands; 2 Department of Orthopaedic Surgery and Traumatology, OLVG Hospital, Amsterdam 1091, AC, The Netherlands; 3 Department of Orthopaedic Surgery and Traumatology, University Medical Center Utrecht, Utrecht 3584, CX, The Netherlands; 4 Department of Epidemiology and Biostatistics and the EMGO Institute for Health and Care Research, VU University Medical Center, Amsterdam 1081, BT, The Netherlands

**Keywords:** Shoulder, PROM, Interpretation, MIC, SDC, DASH, Simple shoulder test, Oxford shoulder score

## Abstract

**Background:**

There is a need for better interpretation of orthopedic treatment effects. Patient-reported outcome measures (PROMs) are already commonly used for patient evaluation. PROMs can be used to determine treatment effects in research as well as in clinical settings by calculating change scores, with pre- and post-treatment evaluation. The smallest detectable change (SDC) and minimal important change (MIC) are two important benchmarks for interpreting these change scores. The purpose was to determine the SDC and the MIC for four commonly used shoulder-related PROMs: Simple Shoulder Test (SST), Disabilities of the Arm, Shoulder and Hand (DASH and *Quick*DASH), and the Oxford Shoulder Score (OSS).

**Methods:**

A cohort of 164 consecutive patients with shoulder problems visiting an orthopedic outpatient clinic completed the SST, DASH, and the OSS at their first visit and 6 months after operative or non-operative treatment. The SDC was calculated with a test re-test protocol (0–2 weeks). For the MIC, change scores (0–6 months of evaluation) were calculated in seven subgroups of patients, according to an additional self-administered ranking of change over time (anchor-based mean change technique). The MIC is defined as the average score of the ‘slightly improved’ group according to the anchor. The *Quick*DASH was computed from the DASH.

**Results:**

The SDC of the SST was 2.8, DASH 16.3, *Quick*DASH 17.1, and OSS 6.0. The MIC change score for the SST was 2.2, DASH 12.4, *Quick*DASH 13.4, and OSS 6.0.

**Conclusion:**

This study shows that on an individual patient-based level, when taking into account SDC and MIC, the change score should exceed 2.8 points for the SST, 16.3 points for the DASH, 17.1 points for the *Quick*DASH, and 6.0 points for the OSS to have a clinically relevant change on a PROM, which is not due to measurement error.

## Introduction

Shoulder pain is the third most common musculoskeletal complaint, after back and knee pains [1]. It is associated with considerable disability for the patient and costs to society. Depending on the diagnosis, many different surgical and non-surgical treatment modalities have been described. In research and clinical practice, determining whether a treatment results in meaningful improvement of symptoms requires the use of high-quality measurement tools.

Over the past decade, there has been a shift in interest from pathophysiological measurements to measuring patient-perceived health. This has resulted in increased use of patient-reported outcome measures (PROMs, also known as PROs). PROMs are self-evaluated measurements of any aspect of a patient's health status, without interpretation of the patient's response by a clinician or anyone else [2]. PROMs are often questionnaires specifically evaluating pain and function from the patient's perspective. The quality of a PROM can be determined by assessing the measurement properties of the instrument. The consensus-based standards for the selection of health measurement instruments (COSMIN) initiative provide a checklist of standards for assessing the measurement properties of validity, reliability, and responsiveness [3,4]. This list does not include interpretability, which is a very important attribute of a questionnaire used in daily clinical practice. Interpretability refers to what a PROM score means; for example, a given score can be interpreted by providing reference data from the general population.

Interpretability is also important in regard to change scores; it is important to know when it can be said that a patient has improved. With many PROMs, change scores are often difficult or impossible to interpret, simply because we do not know exactly what a given difference in score means. Interpreting change in PROM scores requires two benchmarks: the measurement error, expressed as the smallest detectable change (SDC), and the minimal important change (MIC). The SDC is a measure of the variation in a scale due to measurement error. Thus, a change score can only be considered to represent a real change if it is larger than the SDC. The SDC is also known as the minimal detectable change; when using its 95% confidence interval, it can be abbreviated as MDC95%.

The MIC is defined as the smallest measured change score that patients perceive to be important [5-7]. If the SDC is smaller than the MIC, it is possible to distinguish a clinically important change from measurement error with a large amount of certainty. However, this is much more difficult if the SDC is larger than the MIC, since there is a considerable chance that the observed change is caused by measurement error [8]. The MIC is also known as the minimal clinically important difference (MCID).

Both the SDC and MIC are expressed using the same units as the original measure, and thus, these numbers have considerable value for clinical use. Using these two benchmarks to interpret change scores is particularly beneficial when PROMs are applied in individual patients, such as in clinical practice. On a group level, knowledge on the MIC will also provide clinicians with better options for interpreting study results. The MIC can be used to calculate the percentage of patients who report a change greater than the MIC (responders) in each arm of a trial, and these percentages of responders can be compared [9]. Researchers can also use the SDC and the MIC on a group level to calculate an adequate sample size or to perform power analyses, as described by Terwee et al. [8].

Some studies have already assessed measurement error (SDC) and interpretability (MIC) of body part-specific PROMs for patients with shoulder problems [10-18]. The present study aimed to determine the SDC and MIC of four commonly used shoulder PROMs: the Disabilities of the Arm, Shoulder, and Hand (DASH); the Shortened Disabilities of the Arm, Shoulder, and Hand Questionnaire (*Quick*DASH); the Simple Shoulder Test (SST); and the Oxford Shoulder Score (OSS), and compare the results.

## Materials and methods

A prospective cohort of patients with shoulder complaints was consecutively recruited between February 2009 and December 2011 by one orthopedic surgeon (W.J.W.) at the orthopedic outpatient clinic of the Onze Lieve Vrouwe Gasthuis (OLVG), Amsterdam, The Netherlands. Inclusion criteria were age of 16 years or older and the presence of shoulder problems as diagnosed by the orthopedic surgeon (W.J.W.). Both surgical and non-surgical patients were included. Exclusion criteria were fractures, frozen shoulder, and problems with reading and understanding the Dutch language. Institutional approval was obtained by our local ethical committee (OLVG), and written informed consent was obtained from all participants.

### Measurements

Using a web-based system at home, the patients completed an online questionnaire containing the four different body part-specific PROMs at three different time-points: T1 (baseline), T2 (2 weeks after baseline), and T3 (6-month follow-up). The given questionnaires were identical at all three time-points, except for two anchor questions added at T3 (see ‘Outcome measures’ for details). The whole cohort was invited to complete the questionnaire at time-points T1 and T3, whereas only a subset of the cohort was also asked to complete the questionnaire at time-point T2; this was done to limit the response burden. According to international guidelines, a minimum of 50 patients is considered adequate for assessing measurement properties [19]. Since the risk of participant loss to follow-up increased after several months, we included at least 150 patients at baseline. The subset for T2, used to determine the measurement error, was predetermined at 100 patients as recommended by the COSMIN guidelines [19]. The online questionnaire required an answer for each question, such that there could not be any missing values.

### Outcome measures

#### *Simple shoulder test*

The SST measures functional limitations of the affected shoulder in patients with shoulder dysfunction [20,21]. It was originally developed in the USA by Matsen et al. for evaluating patients with common shoulder problems. The SST consists of 12 questions with dichotomous response options; for each question, patients indicate if they are able or unable to perform an activity. The scores of the questions are summarized, with the total score ranging from 0 (worst) to 12 (excellent). The SST has been validated in patients with shoulder complaints [22,23], including Dutch shoulder patients [24].

#### *Disabilities of the arm, shoulder, and hand*

The DASH was developed in the USA by Hudak et al. [25,26]. It is a 30-item, patient-reported questionnaire designed to measure physical functioning and symptoms in people with musculoskeletal disorders of the upper limbs [25]. Items are summarized into a total score, ranging from 0 (excellent) to 100 (worst). The measurement properties have been assessed in patients with disorders of the shoulder, elbow, wrist, and hand [27]. The recent review by Desai et al. [28] showed that the DASH is reliable, valid, and responsive in patients with shoulder disability, and this instrument has been validated in Dutch patients with an upper limb disorder [29].

#### *Shortened disabilities of the arm, shoulder, and hand questionnaire*

The *Quick*DASH is the short version of the original DASH. It was developed by Beaton et al. [26,30]; it contains 11 of the original 30 items, and the score range is from 0 (excellent) to 100 (worst). The measurement properties are comparable with the DASH and have been evaluated in patients with upper extremity disorders. Here, we computed the *Quick*DASH score from the responses to the full DASH questionnaire.

#### *Oxford shoulder score*

The OSS was developed in Oxford (UK) by Dawson et al. [31] for patients with shoulder problems. It contains 12 items related to pain and shoulder function. There are five response options for each question, corresponding to a score ranging from 1 (least difficult) to 5 (most difficult). Scores of the 12 questions are summarized into a total score that ranges from 12 (excellent) to 60 (worst). The OSS has been validated in patients with shoulder complaints [28,31,32], including in Dutch shoulder patients [33].

#### *Anchors*

An anchor is a global rating scale in which patients are asked, in a single question at follow-up, to indicate how much their function (functional anchor) or pain (pain anchor) has changed since baseline [5,34,35]. The response options are as follows: completely recovered, much improved, slightly improved, unchanged, slightly worse, much worse, and worse than ever (see also Tables 1 and 2).

**Table 1:** Mean change score of the four PROMs according to functional anchor

**Functional anchor**		**Mean change score (SD)**			
	***n***	**SST**	**DASH**	***Quick*** **DASH**	**OSS**
Completely recovered	14	2.9 (1.8)	-13.2 (9.6)	-13.5 (11.6)	-6.0 (4.6)
Much improved	37	2.9 (2.2)	-15.6 (13.3)	-17.9 (15.0)	-7.2 (6.8)
*Slightly improved*	*23*	*2.2 (2.7)*	*-12.4 (11.7)*	*-13.4 (12.7)*	*-6.0 (5.3)*
Unchanged	43	-0.1 (1.5)	0.3 ( 9.6)	-0.1 (10.8)	-1.0 (4.2)
Slightly worse	5	-0.6 (0.9)	9.8 (6.2)	6.8 (3.6)	4.2 (4.6)
Much worse	6	-4.0 (2.8)	13.1 (9.6)	14.8 (10.3)	8.3 (5.4)
Worse than ever	0				

The slightly improved group (in italics) was used for the MIC calculation in the present study.

**Table 2:** Mean change score of the Oxford Shoulder Score according to the pain anchor

**Pain anchor**		**Mean change score (SD)**
	***n***	**OSS**
Completely recovered	15	-4.7 (4.2)
Much improved	40	-7.4 (6.8)
*Slightly improved*	*22*	*-4.7 (6.1)*
Unchanged	39	-1.6 (3.9)
Slightly worse	7	4.6 (4.5)
Much worse	5	8.2 (6.1)
Worse than ever	0	

The slightly improved group (in italics) was used for the MIC calculation in the present study.

**Specific instruction to the patients** Try to remember how painful and how limited your shoulder function was before the surgery or, if you were not operated, compared to your initial visit to the outpatient clinic 6 months ago.

**The anchor question—pain** How has the pain of your shoulder changed compared to the first time you completed this questionnaire?

**The anchor question—function** How has the functioning of your shoulder changed compared to the first time you completed this questionnaire?

### Statistical analysis

#### *Smallest detectable change (measurement error)*

Measurement error is the systematic and random error of a patient's score that is not attributed to true changes in the measured construct [5,36,37]. Data from T1 and T2 were used to determine the measurement error. We assumed that there would be no real change in a patient's functioning within a 2-week interval (range, 1 to 4 weeks). Stratford et al. presented the importance that the change scores should be normally distributed and close to zero [38]. Measurement error can be expressed as the standard error of measurement (SEM) or the SDC. The SEM represents the standard deviation of repeated measures in one patient, and was calculated from the square root of the error variance of the ICC (√VarError). The ICC was calculated with a two-way mixed effects model for absolute agreement. The SDC represents the minimal change that a patient must show on the scale to ensure that the observed change is real and not just measurement error. The SDC was calculated as 1.96 × √2 × SEM, and the confidence interval (CI) was calculated [39,40]. These values were expressed in the unit of measurement of the PROM scale.

#### *Minimal important change*

The change scores on the questionnaires were calculated by subtracting each patient's T3 (6 month) score from the T1 (baseline) score and were then used to determine the MIC using an anchor-based mean change score technique [25,41]. The anchor scores were used to categorize patients into seven subgroups, varying from completely recovered to worse than ever. Change scores were calculated in each of the seven subgroups. The MIC was defined as the mean change score in the subcategory of patients who were ‘slightly improved’ according to the anchor scores, and the CI was calculated [5,25]. The SST, DASH, and *Quick*DASH primarily assess shoulder function; therefore, we compared these change scores only to the functional anchor. The OSS includes questions on both pain and function; therefore, we compared the OSS change score with both the pain and functional anchors. We chose to evaluate the patients specifically at 6 months (T3) to have a sufficient number of patients who indicated to be ‘a little better’ to determine the MIC. If you wait too long (especially after surgery), almost all patients will indicate to be ‘a good deal better’ or ‘have no shoulder limitation at all.’

## Results

**Figure 1:**
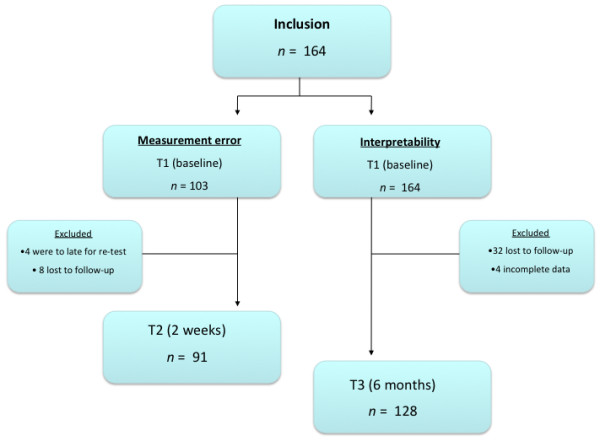
**Flow chart.***n*, the number of patients evaluated.

Figure 1 illustrates the flow of the patients through the study. We asked 164 consecutive patients with shoulder complaints to participate in this study. None refused to participate; thus, the initial response rate at T1 was 100%. Of these, 103 patients were sent the questionnaire at T2. A total of 95 completed the questionnaire at T2; however, only 91 of these could be analyzed since four patients submitted this questionnaire after the maximum period of 4 weeks (response rate for measurement error, 89%). Of all 164 patients, 132 patients completed the questionnaire at T3 (6-month follow-up). Of these, 128 could be analyzed since four patients did not answer the anchor questions on function and pain (response rate for interpretability, 78%). The demographic data are presented in Table 3. At the 6-month evaluation, 53% of the patients were treated surgically.

**Table 3:** Demographic data

		**Baseline**	**SDC analysis**	**MIC analysis**
Number		164	91	128
Mean age, year (range)		41 (16–76)	39 (16–76)	39 (16–76)
Gender	M	115 (70)	62 (68)	59 (69)
	F	49 (30)	29 (32)	39 (31)
Side	L	59 (36)	29 (32)	45 (35)
	R	101 (62)	62 (68)	81 (63)
	B	4 (2)	-	2 (2)
Diagnosis	Impingement syndrome	18 (11)	11 (12)	14 (11)
	Rotator cuff tear	39 (24)	21 (23)	29 (23)
	SLAP lesion	25 (15)	15 (17)	21 (16)
	Anterior instability	75 (46)	41 (45)	58 (45)
	Tendinitis biceps	7 (4)	3 (3)	6 (5)

Data given as numbers (percentages), unless otherwise stated. *M* male, *F* female, *L* left, *R* right, *B* both, *SLAP* superior labral tear from anterior to posterior.

### Smallest detectable change (measurement error)

The 91 patients, who completed questionnaires at both T1 and T2, did so within a mean time period of 12.8 days (SD, 5.5). The ICC was 0.86 for the SST, 0.83 for the DASH, 0.85 for the *Quick*DASH, and 0.90 for the OSS. Table 4 shows the raw scores from T1 to T2 and the change scores. The change scores were normally distributed and close to zero. The SDC was 2.8 for the SST, 16.3 for the DASH, 17.1 for the *Quick*DASH, and 6.0 for the OSS (see Table 4).

**Table 4:** PROM characteristics and scores at baseline and follow-up

	**SST**	**DASH**	***Quick*** **DASH**	**OSS**
Minimum score	0 (worst)	0 (excellent)	0 (excellent)	12 (excellent)
Maximum score	12 (excellent)	100 (worst)	100 (worst)	60 (worst)
T1, mean (SD)	8.5 (2.8)	24.4 (16.0)	25.5 (17.4)	24.4 (7.5)
T2, mean (SD)	8.7 (2.7)	22.5 (14.9)	23.9 (16.1)	23.4 (7.2)
T3, mean (SD)	9.8 (2.5)	16.9 (13.9)	17.1 (14.7)	20.8 (6.5)
Change score T1-T2, mean (SD)	0.01 (1.4)	-0.7 (8.3)	-0.17 (8.7)	-0.6 (3.1)
Change score T1-T3, mean (SD)	1.3 (2.7)	-6.9 (13.8)	-7.9 (15.3)	-3.5 (6.6)
SDC (95%CI)	2.8 (2.8–2.8)	16.3 (14.3–16.7)	17.1 (16.5–17.1)	6.0 (5.6–7.6)
MIC (95%CI)				
Functional anchor	2.2 (1.1–3.4)	12.4 (7.3–17.5)	13.4 (8.0–18.1)	6.0 (3.7–8.3)
Pain anchor				4.7 (2.0–7.4)

*T1* baseline, *T2* 2 weeks, *T3* 6 months, *SDC* smallest detectable change, *MIC* minimal important change, *CI* confidence interval.

### Minimal important change

The mean change scores per subgroup based on the functional and pain anchors are presented in Tables 1 and 2, respectively. From these data, we used the mean change score of the slightly improved group to determine the MIC. The MIC for function was 2.2 for the SST, 12.4 for the DASH, 13.4 for the *Quick*DASH, and 6.0 for the OSS. The MIC for pain was only calculated for the OSS and was 4.7. The MIC data are presented in Table 4.

## Discussion

Monitoring the effects of treatment is of well-recognized importance and is the foundation of modern evidence-based health care. SDC and MIC can be used as benchmarks for the interpretability of a PROM to determine whether the observed change is beneficial to the patients. Here, we determined the SDC and MIC of four commonly used shoulder PROMs in a heterogeneous group of shoulder patients. We found an SDC of 2.8 and a MIC of 2.2 for the SST, an SDC of 16.3 and a MIC of 12.4 for the DASH, and an SDC of 17.1 and a MIC of 13.4 for the *Quick*DASH. For the OSS, we found an SDC of 6.0 and MIC values of 6.0 and 4.7 for function and pain, respectively. Overall, the SDC was slightly larger than the MIC for all four PROMs.

To determine whether a change score on an individual patient level is clinically important and not just measurement error, the SDC score must not exceed the MIC change score [8]. In our study, all PROMs had an SDC that was slightly larger than the MIC. This means that if an individual patient has a change score as large as the MIC, we cannot be 95% sure that this change is not due to measurement error. In other words, the risk of measurement error is larger than 5%, and individual patient's change scores should be interpreted with caution. However, as the differences between the SDC and the MIC were rather small, we think that these four PROMs are suitable for use in clinical practice. In research, the measurement error is much less problematic because group mean changes are analyzed, and the SDC of a mean change is equal to SDC/√*n*. In research, the MIC can also be used to calculate the percentage of patients who report a change greater than the MIC (responders) in each arm of a trial, and these percentages of responders can be compared [9].

Although the observed differences between SDC and MIC were very small, it is desirable to find ways to minimize the SDC. One way of decreasing the SDC in a clinical setting is by averaging multiple measurements (i.e., repeated measurements at one point in time) in order to decrease the measurement error. However, this is difficult using questionnaires because it is a burden for patients and there is a high risk of recall bias. It might also be possible to improve the quality of the questionnaires by adding extra questions or improving the wording of questions.

The observed difference between SDC and MIC is less problematic in research because mean scores of groups of patients are used instead of individual patient scores; therefore, the measurement error should be calculated for a mean score instead of a single score. The SDC of a mean score is much smaller (by a factor of the square root of the sample size) than the SDC of a single score [5,37].

Table 5 presents an overview of the previously reported measurement error (SDC) and MIC of the PROMs evaluated in this paper [10-18]. Our results for the SST are comparable with the results published by Roy et al. [18] with a MIC of 3.0 6 months after shoulder arthroplasty and by Tashjian et al. [15] who determined the MIC in 81 patients with rotator cuff tears. Although Tashjian et al. used a comparable anchor-based mean change score method, they determined the MIC by subtracting the change score of the ‘unchanged group’ from that of the slightly improved group (MIC - _substract_). While there is no consensus on whether this subtraction should be performed, Hays et al. [42] have argued that if the mean change in the unchanged group is 2 points and the mean change in the slightly improved group is 4 points, this means that a 2-point change is insufficient and that it takes a greater change of 4 points to constitute a MIC [42]. We agree with Hays et al. [42] that the unchanged change score should not be subtracted from the slightly improved change score. However, it is possible to calculate the MIC - _substract_ from our data (see Tables 1 and 2). For example, for the SST, the MIC - _substract_ for the functional anchor would be -2.2 - -0.1 = -2.1 and for the OSS -6.0 - -1.0 = -5.0. Both techniques give almost the same MIC values for the SST, DASH, and *Quick*DASH, only for the OSS there is a small difference.

**Table 5:** **Overview of previously published SDC and MIC values for the SST, DASH, *Quick*DASH, and OSS**

**PROMs**	**Study**	**Number**	**SDC**	**MIC**
SST	Tashjian et al. [15]	81	n.m.	2.8
	Roy et al. [18]	120	-	3.0
DASH	Schmitt and Di Fabio [14]^a^	53	14.6^a^	10.2
	Beaton et al. [10]	361	10.7	11.5
	Gummesson et al. [11]	109	n.m.	10
	Gabel et al. [16]	41	7.9	-
*Quick*DASH	Mintken et al. [12]^a,b^	101	13.3^a,b^	8.2
	Polson et al. [13]^c^	35	n.m.	13.1^c^
	Gabel et al. [17]^a^	47	18.6^a^	
OSS	-	-	-	-

*SDC* smallest detectable change, *MIC* minimal important change, *n.m.* not mentioned. ^a^Data recalculated to a 95% interval. ^b^Mintken used the unchanged group at follow-up and test re-test. ^c^Polson used the much improved group for the MIC calculation; we used the minimally improved group in this table.

Our results for the DASH were comparable with the results found in the literature. Schmitt and Di Fabio [14] used the anchor-based mean change method to analyze a heterogeneous group of 53 shoulder patients and found a SEM of 5.22 and a MIC of 10.2. They used a 90% interval for the SDC calculation. To improve comparability, we recalculated their data to a 95% interval, resulting in an SDC of 14.6. Beaton et al. [10] studied a cohort of 361 heterogeneous shoulder patients treated by physiotherapists, using a comparable anchor-based mean change method; they found an SEM of 3.9, an SDC of 10.7, and a MIC of 11.5. Gummesson [11] found a MIC of 10 in a comparable study in 109 upper extremity patients. Gabel et al. [16] found a lower SDC of 7.9; this is probably due to the fact that the test re-test was done within 48 h, increasing the chance of a recall bias.

The results of the *Quick*DASH were also comparable with those in the current literature. Mintken et al. [12] analyzed 101 shoulder patients. Using a comparable anchor-based technique, they found a MIC of 8.2. They calculated SDC using the unchanged group at follow-up, which is a suboptimal technique for determining the measurement error because of the risk of bias due to the lack of validity of the anchor [43]. They also used a 90% interval for the SDC calculations; we recalculated the SDC to a 95% interval, resulting in an SDC of 13.3. Polson et al. [13] analyzed 35 upper extremity patients with an anchor-based mean change technique. They found a higher MIC of 19 points, most likely because they used the ‘much improved’ group for the MIC calculations instead of the slightly improved group as we did in this study. Polson et al. [13] also reported the change score of the slightly improved group to be 13.1; this information is used in Table 5 to improve the comparability of our results. Gabel et al. found comparable results to our study, with a 95% recalculated interval of the SDC of 18.6 for the *Quick*DASH [17]. There is no international consensus on the optimal cut-off point on an anchor; however, we think that the slightly improved group best reflects a minimal important change opposed to the much improved group.

Our method to calculate the MIC is comparable with Jaeschke et al. and Redelmeier and Lorig [6,7]. Jaeschke et al. used a 15-point rating scale and used the mean change in patients who reported to be ‘almost the same,’ ‘a little better or a little worse,’ or ‘somewhat better or somewhat worse’ as the MIC value. Redelmeier used a similar 15-point scale and used the mean change in patients who reported to be ‘a little better or a little worse’. We used a 7-point rating scale and used the change in patients who reported to be slightly improved as the MIC.

To the best of our knowledge, there is no previous data on SDC and MIC for the Oxford Shoulder Score [44]. One-third of the questions in the OSS are pain related, so we used both anchors. We found an SDC of 6.0 points on a scale from 12 to 60. The MIC was 6.0 corresponding to the functional anchor and 4.7 to the pain anchor.

Strengths of this study are that there were almost no missing data and we had very high response rates at all time-points. This is a clear advantage of web-based questionnaire administration. Furthermore, we included twice the recommended minimal number of patients.

There are several limitations to our study. First, we used a heterogeneous population for calculation of the MIC. There is no evidence in the literature that the MIC differs among (sub)populations of different diagnosis and surgical or non-surgical treatment, but it has been suggested that this should be evaluated [35,45]. This was not possible in our study because the subgroups would be too small. The advantage of using a heterogenic cohort is that it provides a MIC estimation that can be used in all kinds of shoulder disorders and for surgical and non-surgical treatments. Future studies should examine if and how the MIC varies among subgroups. Second, our patients had to complete three different PROMs at the same time. This could be a response burden to the patient, which might lead to loss of interest during completion. Theoretically, this could result in increased measurement error and a higher SDC. Third, we computed the *Quick*DASH from the full DASH questionnaire. This is not the same as completing the *Quick*DASH questionnaire independently. Fourth, the test-retest was determined within 1–4 weeks (average 12.8 days). We cannot be completely sure that none of the patients changed within this time frame. However, in The Netherlands, patients start physiotherapy treatment in general not earlier that 1–2 weeks after their initial visit and none of the patients were treated surgically within the test re-test period, so we do not expect patients to change within this time frame. Fifth, although anchor-based techniques are considered the best method for assessing the MIC [35]; there is a debate in the literature about the validity of anchors and the best statistical approach for calculating the MIC [46]. For example, a disadvantage of the mean change method is that it uses only the average change score of one patient subgroup for the MIC calculation, meaning that only 23 patients determined the MIC value in this study. For these methodological reasons, it has been recommended that the MIC of PROMs should be determined in multiple studies [47]. Our study therefore contributes to a better understanding of the change scores of PROMs in shoulder patients.

## Conclusion

This study shows that on an individual patient-based level, when taking into account the SDC and MIC, the change score should be above 2.8 points for the SST, above 16.3 points for the DASH, above 17.1 points for the *Quick*DASH, and above 6.0 points for the OSS to show a relevant change that is not due to measurement error.

## Competing interests

The authors declare that they have no competing interests.

## Authors’ contributions

DAvK designed the study and wrote the protocol. He managed the database and preformed the analysis. He wrote and revised the manuscript. WJW helped in designing the study and included all the patients at the outpatient clinic. He critically reviewed the study. LWAHvB helped with the database management and performed parts of the analysis. She critically reviewed the manuscript. VABS helped in designing the study and performed parts of the analysis. She critically reviewed the manuscript. RMC helped in designing the study. He critically reviewed the manuscript. CBT helped in designing the study, advised on the statistical analysis, and critically reviewed the manuscript. All authors read and approved the final manuscript.
